# A Systematic Review of Carotenoids in the Management of Diabetic Retinopathy

**DOI:** 10.3390/nu13072441

**Published:** 2021-07-16

**Authors:** Drake W. Lem, Dennis L. Gierhart, Pinakin Gunvant Davey

**Affiliations:** 1 College of Optometry, Western University of Health Sciences, 309 E Second St, Pomona, CA 91766, USA; drake.lem@westernu.edu; 2 ZeaVision, LLC, Chesterfield, MO 63005, USA; dgierhart@zeavision.com

**Keywords:** diabetic retinopathy, macular xanthophylls, carotenoids, macular pigment, macular pigment optical density, MPOD, lutein, zeaxanthin, *meso*-zeaxanthin, diabetes, diabetic retinopathy, retinal neurodegeneration, neuroprotection

## Abstract

Diabetic retinopathy, which was primarily regarded as a microvascular disease, is the leading cause of irreversible blindness worldwide. With obesity at epidemic proportions, diabetes-related ocular problems are exponentially increasing in the developed world. Oxidative stress due to hyperglycemic states and its associated inflammation is one of the pathological mechanisms which leads to depletion of endogenous antioxidants in retina in a diabetic patient. This contributes to a cascade of events that finally leads to retinal neurodegeneration and irreversible vision loss. The xanthophylls lutein and zeaxanthin are known to promote retinal health, improve visual function in retinal diseases such as age-related macular degeneration that has oxidative damage central in its etiopathogenesis. Thus, it can be hypothesized that dietary supplements with xanthophylls that are potent antioxidants may regenerate the compromised antioxidant capacity as a consequence of the diabetic state, therefore ultimately promoting retinal health and visual improvement. We performed a comprehensive literature review of the National Library of Medicine and Web of Science databases, resulting in 341 publications meeting search criteria, of which, 18 were found eligible for inclusion in this review. Lutein and zeaxanthin demonstrated significant protection against capillary cell degeneration and hyperglycemia-induced changes in retinal vasculature. Observational studies indicate that depletion of xanthophyll carotenoids in the macula may represent a novel feature of DR, specifically in patients with type 2 or poorly managed type 1 diabetes. Meanwhile, early interventional trials with dietary carotenoid supplementation show promise in improving their levels in serum and macular pigments concomitant with benefits in visual performance. These findings provide a strong molecular basis and a line of evidence that suggests carotenoid vitamin therapy may offer enhanced neuroprotective effects with therapeutic potential to function as an adjunct nutraceutical strategy for management of diabetic retinopathy.

## 1. Introduction

Although half a billion individuals are estimated to be living with this condition globally, diabetes remains severely underdiagnosed, with one in every two individuals living with the disease unaware [1,2,3]. It is further projected that the prevalence of diabetes is likely to increase to 700 million by the year 2045 [2,3,4]. The systemic disease of endocrine origin leads to progressive damage throughout the body with all end-organs suffering damage [5,6,7,8,9,10]. Chronic hyperglycemia causes irreversible damage to all parts of the eye. Both the anterior segment structures, cornea, conjunctiva, and lens as well as the posterior segment become damaged [6,11,12]. In the posterior segment, particularly the retina in an individual shows pathognomonic damage, leading to diabetic retinopathy (DR) [6,7,11,12]. The prevalence of diabetes mellitus (DM) has reached epidemic proportions [4,12]. Increased life expectancy and the chronic nature of diabetes with no “true” cure has led to and will continue being a massive health care and socio-economic burden [2,3,5,13,14]. Consequently, it is expected that annual global expenditures will exceed USD 825 billion by the year 2030 [15].

The natural history of DR features retinal capillary degeneration and subsequent significant visual impairment [16], when poorly managed, causes vasoproliferative disease in retina and/or edema in the central macular region; these complications may arise consecutively or simultaneously [11,12]. Approximately one in three individuals with diabetes is affected by retinopathy [4,5,6,7]. The severity of DR is associated with both with the duration of diabetes and glycemic control [17,18]. An estimated 4.1 million individuals in the US are afflicted with DR, of which approximately 899,000 have vision-threatening retinopathy [1]. It is estimated globally that 146 million adults have DR with a projected increase to 191 million by 2030 [2,3,14]. The vision loss due to hyperglycemia-induced retinopathy is irreversible as the retinal tissue does not regenerate. However, the damage due to diabetes and DR is preventable, and thus allows for a potential of improvement in the quality of life, decrease in susceptibility to further complications, and reducing health care expenditures [4,7].

Hyperglycemia-induced damage to other parts of the body has been shown to correlate with the severity of DR, including peripheral neuropathy, nephropathy and cardiovascular complications [5,6,7,8,9,10]. It is well known that chronic hyperglycemic states promote oxidative damage particularly in highly susceptible regions with corresponding high metabolic demands. The extremely metabolically active retinal tissue is particularly susceptible to oxidative damage due to constant exposure to light [19,20]. Recent work strongly implicate that neurodegeneration in retina is proliferated by pro-oxidative and pro-inflammatory mechanisms prior to indications of clinical retinopathy [5,7,10,18,20,21,22,23,24]. Inherent defense mechanisms against oxidative damage in the retina involve constant neutralization of reactive oxygen species (ROS). Congruously, both endogenous and exogenous antioxidants are essential in maintaining cellular redox homeostasis [20,25,26]. Quite appropriately, it is postulated that the interdependence between prolonged hyperglycemia, oxidative stress, and changes in redox homeostasis is a key factor contributing to the pathogenesis of diabetic retinopathy [19,25].

**Figure 1:**
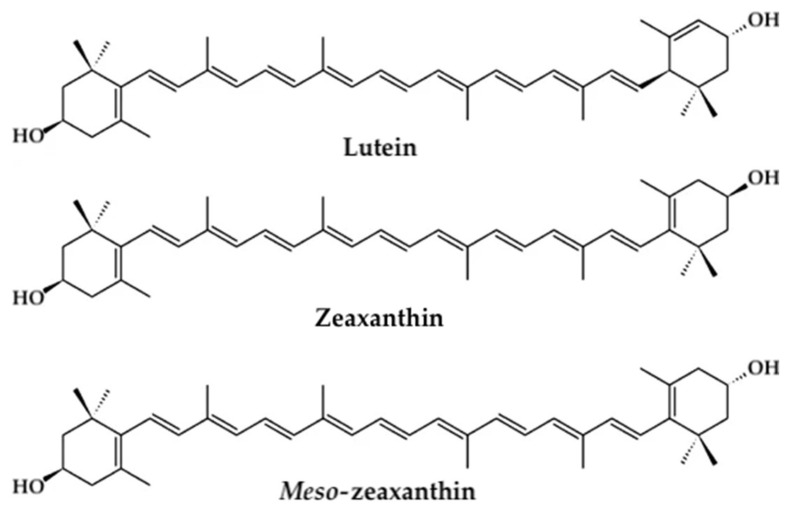
Chemical structures of isomeric xanthophyll carotenoids lutein, zeaxanthin, and *meso*-zeaxanthin.

More than 750 naturally occurring phytochemical carotenoids have been identified and characterized, of which, approximately 20 types are present in serum and tissue [27,28,29,30]. Among them, the only dietary carotenoids which accumulate in the human eye are lutein and zeaxanthin [27,30]. They belong to the xanthophyll class of carotenoids which contain oxygen in their polyene chain structure and are more lipophilic in comparison with the other subgroup of carotenoids known as carotenes, which do not contain oxygen and are purely hydrocarbons [27,31]. Three isomeric xanthophyll carotenoids—lutein, zeaxanthin, and *meso*-zeaxanthin (Figure 1)—are believed to possess significant antioxidant and anti-inflammatory properties in the retina and have been shown to benefit in prevention of age-related macular degeneration (AMD) [25,27,32,33,34,35]. Oxidative insult contributing to retinal neurodegeneration is common to the pathogenesis of both DR and AMD. Hence, it is hypothesized that xanthophyll carotenoids may be clinically beneficial in management of DR.

To the best of our knowledge, the neuroprotective potential afforded by these xanthophylls in clinical management of DR has not been thoroughly reviewed. The primary objective of this systematic review focuses on summarizing the evidence from animal models, clinical observational studies, and randomized controlled trials that have reported on the putative relationship between DR and carotenoids lutein, zeaxanthin, and/or *meso*-zeaxanthin. Thus, the goal of this systematic review is to determine the degree of clinical benefits of carotenoids as an adjunct therapy for the management of DR.

### Retinal Changes in Diabetics

Retinal changes in diabetes are graded by fundoscopic lesions as outlined by the International Clinical Disease Severity Scale [12,13,16,36,37,38]. Large-scale clinical trials established the severity classification system (Table 1) that is currently used: The Early Treatment Diabetic Retinopathy Study (ETDRS) and the Wisconsin Epidemiological Study of DR (WESDR) [16,37,38,39]. Non-proliferative diabetic retinopathy (NPDR) is seen as microvascular abnormalities limited to the retinal surface. Additionally, some other features visible are intraretinal hemorrhages (“dot and blot” shaped), microaneurysms, hard exudates, and intraretinal microvascular abnormalities (i.e., tortuous sinus shunt vessels) [37,38,39]. The degeneration of capillaries and apoptosis in the endothelium are an outcome of progressive oxidative damage in this stage that leads to capillary nonperfusion and vascular occlusion leading to retinal ischemia/hypoxia. This compromises oxygenation and further aggravates oxidative and pro-inflammatory processes in the extremely metabolically-active retina [17,18,36]. These events promote angiogenesis due to the release of vascular endothelial growth factor (VEGF) [17,18,36,40]. The manifestation of cotton wool spots represents hypoxic retina that leads to neurodegeneration [18]. Subsequent retinal neovascularization with aberrant angiogenesis marks disease progression to proliferative diabetic retinopathy (PDR). The new blood vessel formation is an ineffectual attempt to re-establish vascular perfusion and restore homeostasis. However, the response mechanism itself paradoxically further threatens function and viability of the retina ensuing leakage or hemorrhaging into the vitreous cavity, which can lead to retinal detachment and irreversible vision loss [12,13,16,36].

Structural and cellular changes to the retinal architecture enhance permeability, con-tributing to the break in the blood–retinal barrier that leads to diabetic macular edema (DME); the primary cause of significant vision loss in DR [17,36]. Signs of overt edema are seen during fundoscopic exam. However, subtle edema, evidenced by thickening of basement membrane and presence of exudates, is best visible using optical coherence tomography (OCT) [41,42]. It is extremely important to note, the onset of DME can occur at any stage of DR [5,36].

## 2. Diabetic Retinopathy and Macular Pigment

### 2.1. Basics of Macular Pigment

The yellow spot that is visible during ophthalmoscopy is due to macular pigment, which contains three carotenoids—(1) lutein, (2) zeaxanthin, and (3) a stereo isomer of zeaxanthin called *meso*-zeaxanthin [43,44]—which are known as macular xanthophylls. They are uniquely concentrated in the fovea centralis. A recent study that used confocal resonance Raman microscopy showed that although both lutein and zeaxanthin are concentrated in the fovea, zeaxanthin mainly accumulates in the inner plexiform, outer plexiform and outer nuclear layers of the retina [43,44,45,46,47]. Lutein is more diffusely distributed throughout the macula and is present at lower concentrations in comparison to zeaxanthin at the fovea [47]. Humans have lost the ability to synthesize lutein and zeaxanthin in vivo and thus lutein and zeaxanthin can only be acquired through dietary intake [27]. Common food sources that can provide these xanthophylls are green leafy cruciferous vegetables and egg yolks [44,48,49,50]. Unless artificially supplemented, *meso*-zeaxanthin found in the retina is an outcome of biochemical conversion of lutein via RPE65 isomerase in the retinal pigment epithelium (RPE) [44,47,48,51,52,53,54]. The biological processes involving the uptake, metabolism, and transport of xanthophyll carotenoids to the retina have been explored in greater depth in these review articles [27,28,44,48,51,53,54,55,56]. Supplementation of macular xanthophylls improves their levels in the serum [44,48,52,57] and is well known to accumulate in the human retina [27,43,57,58,59,60,61,62,63,64,65,66,67,68].

Clinical measurement of the macular pigment optical density (MPOD) is as close as we can get to quantification of macular carotenoids. The level of MPOD is indeed a biomarker and is strongly associated with maintenance of retinal health and optimal visual function in both health and disease [44,46,50,59]. Prior reports have demonstrated that carotenoids afford enhanced protection in the retina, specifically in the central region, via two proposed mechanisms: (1) acting as a naturally occurring blue light filter or blocker, and (2) a potent antioxidant and anti-inflammatory substance in the retina [44,50,59,69,70,71,72]. The short-wavelength (blue) light triggers production of ROS due to photo-oxidation that leads to damage of the lipid bilayer in cell membranes, proteins, and DNA, in addition to mitochondrial dysfunction which leads to cellular necrosis [44,70,71,72,73,74]. Absorption of the blue light by macular pigment prevents formation of ROS and the consequent oxidative injury triggered by photo-oxidation [43,72,73]. These properties of carotenoids in macular pigment may in part explain how MPOD levels provide neuroprotective capabilities in the retina.

### 2.2. Measuring MPOD

There are several techniques available to effectively quantify MPOD in vivo [27,46,50,75,76,77,78,79,80]. The techniques can be broadly divided into two types: (1) subjective—that is, requiring patient response or participation and (2) objective—that is, requiring minimal to no participant involvement to collect measurements [46,50,75,77,81,82,83,84].

Heterochromatic flicker photometry (HFP) is the most widely used technique to measure MPOD [46,50,75,76,78,79]. The precise mechanism used to measure macular pigment levels by HFP devices may vary based on the manufacturer, which has been described in prior literature [27,45,46,50,69,77,78,85,86,87]. Briefly, current HFP devices adjust the intensity of the blue to green ratio in the target stimuli, which is perceived as a flicker. Steady light is observed when the blue component is fully absorbed by the macula, and only green is visible. This is the lowest point in the absorption curve that is measured and converted to MPOD density units [46,50,77,79,80,88,89].

Fundus reflectometry [61,83,90,91,92,93], fundus autofluorescence [81,82,94] and resonance Raman spectroscopy [47,95,96] are all non-invasive, objective imaging modalities that can measure MPOD [50,75]. Details regarding both subjective and objective techniques to measure MPOD can be found in these review articles [27,45,46,50,75,82,94,97,98].

## 3. Materials and Methods

This systematic review was conducted in accordance with the Preferred Reporting Items for Systematic Reviews and Meta-Analysis (PRISMA) reporting guidelines [99].

### 3.1. Literature Search and Selection Strategy

Two authors (PGD and DWL) performed a wide-ranging search of the scientific databases National Library of Medicine and Web of Science to identify all relevant publications reporting on the association between macular carotenoids and DR until 21 December 2020. Under the guidance of the university librarian, the two authors conducted the full search strategy and data collection together using the following keywords and the combination of their variants during the search query: carotenoids, lutein, zeaxanthin, *meso*-zeaxanthin, macular pigment, macular pigment optical density, MPOD, diabetes, diabetic eye disease, and diabetic retinopathy. The database selection strategy was limited to records pertaining to macular carotenoids (i.e., lutein and/or zeaxanthin and/or *meso*-zeaxanthin) and diabetic retinopathy only. Primary search results were identified for initial screening according to titles and abstracts available in English by PGD and DWL. Among the eligible records, full-text publications were retrieved and evaluated for study inclusion or exclusion criteria. To ensure all relevant studies were included in this review, we individually conducted backward and forward searches of the eligible publications by reviewing reference lists and cited references, respectively. All records retrieved in full text were individually screened and evaluated by two authors (PGD and DWL) for inclusion/exclusion and any discrepancies were resolved through discussion involving the third author (DLG). Selected publications were quantitative research articles evaluating the association between MPOD/carotenoids (including lutein and/or zeaxanthin and/or *meso*-zeaxanthin) and diabetic retinopathy. Additional records involving other forms of diabetes-associated ocular disease were not considered in this review (such as diabetic cataract, diabetic anterior segment or corneal changes associated with hyperglycemia). The full inclusion criteria for eligible publications from experimental and clinical studies are outlined below.

### 3.2. Study Selection

Experimental animal studies included in this review met the following criteria: (1) evaluating the effects of treatment with carotenoids (including lutein, L and/or zeaxanthin, Z) on outcomes of retinal neurodegeneration, such as markers of oxidative stress, cell viability and visual performance in murine models of DR; (2) carotenoid interventions include powder diet supplemented with L and/or Z only, nutraceutical diet containing L/Z, and powder diet supplemented with micronutrient formula containing L/Z; (3) presentation of DR pathology induced using standard induction methods (i.e., administration of the drug alloxan/streptozotocin, high-sugar diet, and surgical or chemically-induced damage) or genetic models (namely the *Lepr*^db^ model) in rodents only; and (4) experimental models of type 1 or type 2 diabetes in rodents were included.

Inclusion criteria for this systematic review were: (1) observational studies evaluating the association among macular xanthophylls and DR; (2) prospective randomized clinical trials assessing the benefits of carotenoid vitamin therapy in diabetic patients; (3) interventions include dietary carotenoid supplementation (containing L and/or Z) or in a multivitamin formula containing micronutrients and antioxidants; (4) assessment of macular carotenoid levels reported by serum/plasma concentrations of L/Z, or by validated MPOD measurement techniques; (5) cohorts of diabetic patients (type 1 diabetes mellitus, T1DM; and/or type 2 diabetes mellitus, T2DM); and (6) study cohorts of both T1DM and T2DM with either no retinopathy present or mild/moderate NPDR.

Exclusion criteria were based on the following: (1) carotenoid treatment did not include either lutein and/or zeaxanthin in formulation/design; (2) carotenoid treatment included other types of carotenoids; (3) experimental diabetes pathology (as listed previously) were not standard methods of induction; (4) inclusion of adults with other forms of diabetes associated eye disease; and (5) publications were not available in English.

### 3.3. Data Extraction, Reliability and Risk of Bias Assessment

The PRISMA reporting guidelines were carefully followed as closely as possible, as discussed previously [99]. The risk of bias was assessed using standard metrics established to evaluate the intervention studies and randomized controlled trials. The SYRCLE’s RoB tool which is an adaptation of Cochrane RoB tool was used to evaluate the risk of bias for the animal studies [100]. The Cochrane Collaboration’s tool for assessing risk of bias for the randomized controlled trials [101].

## 4. Results

### 4.1. Search and Selection of Studies

**Figure 2:**
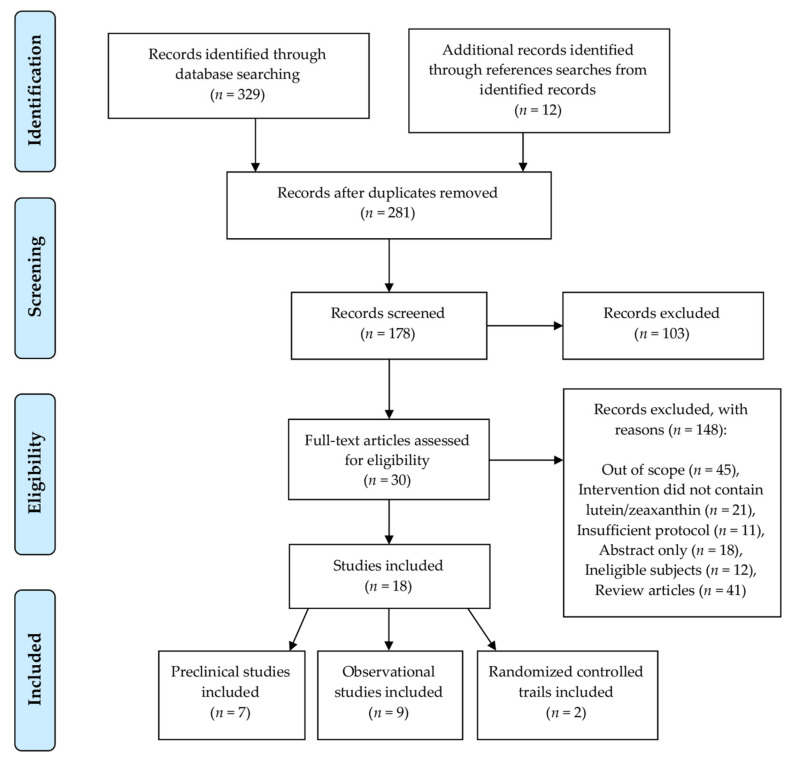
Flow diagram of literature search and selection criteria.

In total, 397 studies were identified during the primary search from scientific databases (Figure 2). After removing duplicate records and including additional records retrieved from reference list searches, 281 studies remained for titles and abstract screening. Consequently, 103 records were excluded based on article type, with an additional 148 records excluded due to the aforementioned inclusion criteria for clinical and preclinical studies. Finally, 30 records were identified to be eligible for full-text assessment, of which, 18 studies were included in the final review: seven preclinical studies [102,103,104,105,106,107,108], nine observational clinical studies [19,25,109,110,111,112,113,114,115] and two interventional clinical trials [34,116].

### 4.2. Carotenoids in the Management of Diabetic Retinopathy—Animal Studies

**Figure 3:**
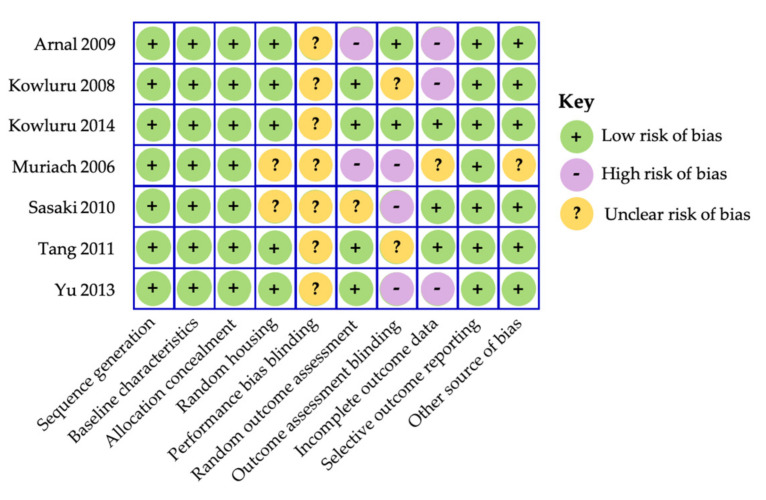
SYRCLE’s risk of bias assessment for animal studies [100].

Figure 3 provides a summary of the assessment of risk of bias using the SYRCLE’s RoB tool [100]. The studies were unclear on performance bias blinding and outcome assessment blinding was not performed (see Figure 3). However, given that studies have utilized laboratory analysis and histology and not psychophysical response measured in animals or subjective interpretations we can overall safely conclude that the overall risk of bias in these studies were low.

There is an increasing amount of research and animal trials that substantiate the neuroprotective effects of carotenoids lutein and zeaxanthin in rodent models of DR using either chemical induction or genetic modes to engender diabetic state (Table 2) [102,103,104,105,106,107,108]. Pharmacological injection of alloxan or streptozotocin (STZ) are often used to recapitulate T1DM pathology in both mice and rats through death of pancreatic beta cells and subsequent insulin deficiency [102,103,104,105,106,117,118,119,120]. Genetic modes offer unique models to examine pathophysiological mechanisms of metabolic perturbations that may contribute to incident retinopathy; in particular, leptin receptor deficient (db/db) mice develop morbid obesity and hypoinsulinemia, making them a desirable model for replicating conditions found in T2DM [107,108,118,119,120]. Importantly, these murine models mimic the characteristic pathological changes induced by hyperglycemia, including oxidative stress driven by free radicals, chronic low-grade inflammation, morphological abnormalities from capillary cell death, and visual dysfunction. Results from these studies are congruous, indicating that lutein and zeaxanthin supplementation has significant potential to protect the retina from the onset of DR.

The importance of macular carotenoid’s antioxidant properties is evident by their enhanced capacity to ameliorate the extent of oxidative injury caused by hyperglycemia in diabetic retina. Supplementation with lutein and/or zeaxanthin was shown to protect against measures of oxidative and nitrosative stress, marked by significant reductions in malondialdehyde, 8-OHdG (oxidatively-modified DNA), and nitrotyrosine, respectively [102,103,105,117,121,122,123]. Additionally, one study found that micronutrients containing carotenoids prevented a significant rise in retinal ROS levels in T1DM rats following treatment with the EyePromise Diabetes and Visual Function Study (DVS) formula (ZeaVision LLC, Chesterfield, MO, USA) [104,124]. These findings suggest that the mechanism of protection against oxidative damage to the retina may involve improving mitochondrial dysfunction, the primary source of aberrant free radical production as a consequence of hyperglycemia [26,125,126,127,128,129]. In fact, lutein and zeaxanthin were shown to protect against mitochondrial stress induced by T1DM pathology, and improved retinal expression of mtDNA-encoded proteins involved in oxidative phosphorylation and mitochondrial biogenesis [26,102,103,108,117]. Thus, dietary treatment using lutein and zeaxanthin supplementation may prevent early lesions of retinopathy by alleviating pro-oxidant stressors and redox imbalance propagated by hyperglycemic state.

Dietary augmentation of the compromised endogenous antioxidant defenses has been considered the key modulator in the pathogenesis of DR. Multiple studies found that lutein and zeaxanthin recovered enzymatic activity and expression levels of glutathione, glutathione peroxidase and manganese superoxide dismutase [102,103,105,107,117]; indicating a reversal of hyperglycemic-induced impairment in free radical detoxification and clearance mechanisms [26,121,130,131]. Similarly, one animal model demonstrated that an AREDS-based micronutrient formulation improved total antioxidant capacity in the retina, as well as metabolic abnormalities associated with early stages of retinopathy progression [104]. By regenerating endogenous antioxidant capacity, dietary supplementation with lutein and zeaxanthin may serve to reduce the proliferation of consequent damage brought on by oxidative stress and inflammation in diabetic retina [104,121,130,131,132,133,134,135,136].

Macular carotenoids may further protect against retinal neurodegeneration by limiting activation of low-grade inflammatory pathways triggered by metabolic and oxidative insults concomitant with hyperglycemic conditions [17,18,21,22,132,133,137,138]. Consistent with this, carotenoid supplementation was shown to mitigate T1DM-induced increase in retinal pro-inflammatory mediators, such as nuclear transcriptional factor-B (NF-kB), interleukin-1β and intercellular adhesion molecule-1 [103,104,105,137,139,140,141,142,143,144,145]. In addition, several studies found that carotenoids demonstrated significant potential to offset pathogenic factor associated with pivotal changes observed in early and advanced stages of retinopathy [17,21,22,104,108,117]; namely, increased cell permeability and neovascularization, respectively [133,135,136,142,146,147]. This neuroprotection following lutein and zeaxanthin administration was evidenced by attenuating the upregulation of pro-angiogenic factor VEGF in diabetic retina of mice and rats [104,108,117]. Preliminary reports suggest carotenoids may protect the local retinal tissue by reducing pro-inflammatory signaling, thereby limiting exacerbation of the inflammatory response to surrounding tissues [138,143,144,148].

The neuroprotective potential of lutein and zeaxanthin positively influencing the pathogenesis of DR was most substantial preventing changes in retinal morphology as a consequence of accelerated capillary cell loss induced by hyperglycemia; regarded as hallmark features of early-stage retinopathy [17,18,148,149,150,151,152,153]. Lutein and zeaxanthin improved cell viability and markedly enhanced cell survival of the retinal vasculature, which was marked by significant reduction in apoptotic nuclei and formation of degenerative (acellular) capillaries [102,103,104,106,154,155]. Similarly, carotenoid treatment completely reversed significant loss of ganglion cells caused by hyperglycemic state in murine model [102,106,107]. Studies found lutein and zeaxanthin effectively protected against DM-induced alterations in retinal histology, such as accelerated thinning of the ganglion cell layer (GCL), inner plexiform layer (IPL), inner nuclear layer (INL), outer nuclear layer (ONL), and the photoreceptor layer (inner and outer segment) [102,106,107]. It is important to note, improvement in the photoreceptor layer indicate that the extent of augmentation in cell survival following lutein and zeaxanthin supplementation can be seen maintaining both vascular and non-vascular cells throughout the retina.

Experimental studies strongly suggest that carotenoids may sufficiently protect against the cumulative effect of hyperglycemic-induced retinopathy, or rather progressive neurodegeneration in retinal function made evident by abnormal or delayed response on electroretinogram (ERG). Studies found that lutein and zeaxanthin preserved measures of inner retinal function at the post-receptor level, attenuating DM-induced reduction in oscillatory potentials and the amplitudes of both a- and b-waves on ERG [102,104,105,106,156,157,158,159]. Increased retinal expression of synaptophysin and brain-derived nuclear factor (BDNF) seem to corroborate these findings, wherein greater synaptic activity and cell survival in the inner retina were observed following supplementation with lutein and zeaxanthin [106,160,161,162,163]. Thus, preliminary findings offer substantial evidence demonstrating neuroprotective effects of macular carotenoids preventing vision loss in models of both type 1 and type 2 diabetic retina.

Although results from these animal models are promising, interpretation of the immediate translative potential for clinical application must be performed with prudence. Briefly, accumulation of carotenoids in the macula is unique to primate retinas, and therefore macular pigments cannot be fully studied using only rodent models of DR [27,164,165]. It is important to note the potential limitations depending on the method of DM-induction utilized in rodents; namely, pathophysiological differences in T1DM (via pharmacological injection with STZ/Alloxan) compared to T2DM (using genetic modes) [118]. For instance, while models of T1DM using STZ are more common since it results in the fastest rate of disease progression, evidence from these reports is not directly comparable between animal models of DR, and therefore each induction method contains its own set of advantages and limitations [118]. In light of this, when accounting for average body weight and daily food consumption in these rodent models, the concentrations of carotenoids and antioxidants used in some reports [104,117] are largely equivalent to the dosage of lutein and zeaxanthin used in clinical intervention trials [34,116,166]. Thus, findings from these preclinical studies are encouraging since the observed protective effects are not due primarily as a consequence of inflated carotenoid concentrations that are beyond clinical relevance for humans. Nonetheless, we can conclude there is a significant and growing body of evidence in agreement with the neuroprotective benefits of lutein and zeaxanthin in ameliorating the onset and progression of hyperglycemia-induced retinopathy.

### 4.3. Clinical Studies Using Carotenoids in the Management of Diabetic Retinopathy

Clinical studies implicate MPOD depletion, as well as low serum levels of lutein and zeaxanthin, may represent a novel clinical feature of DR; one that is likely contingent upon several metabolic perturbations associated with chronic hyperglycemia in type 1 and type 2 diabetes. Reports from observational studies are consistent in demonstrating carotenoid levels (measured both in serum and the macular pigment) are further reduced among diabetic patients with clinically evident retinopathy (Table 3) [19,25,34,109,110,111,112,113,114,115,116]. In fact, one study found that lower plasma concentrations of lutein and zeaxanthin were significantly associated with greater risk of incident maculopathy as well as disease progression in patients with T2DM [109]. Macular pigment data seem to mirror these findings, providing a strong line of evidence that MPOD levels are substantially lower in diabetic retina [34,110,111,112,113,114,115] and in particular, individuals with T2DM with retinopathy [19,25,112]. Several studies have also shown the severity of diabetic maculopathy was significantly associated with lower MPOD levels [110,111,112,113]. Moreover, preliminary findings are largely comparable and suggest that the relationship between compromised macular pigment and incident retinopathy may vary between diabetes types [19,25,112].

**Figure 4:**
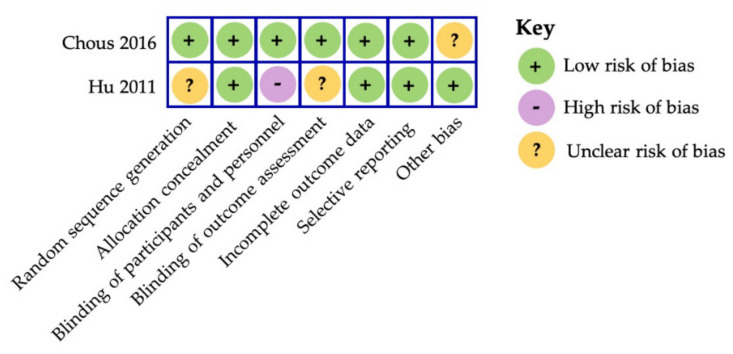
The Cochrane Collaboration’s tool for assessing risk of bias in randomized controlled trials [101].

There is limited evidence of RCTs evaluating the benefits of carotenoids in management of diabetic retinopathy. We used the Cochrane Collaboration’s tool for assessing risk of bias, which covers the following domains—selection bias, performance bias, detection bias, attrition bias, reporting bias, and other bias [101]. Figure 4 provides a summary of the risk assessed using the Cochrane Collaboration’s tool.

It is well known that both type 1 and type 2 diabetic patients with mild NPDR or no retinopathy exhibit a notable range of visual function impairment, even in the absence of clinically relevant lesions of neurodegeneration in the retina [167,168,169,170,171,172,173]. Following active oral supplementation containing lutein and/or zeaxanthin micronutrients, two interventional studies (Table 4) observed marked improvements in serum carotenoids and MPOD levels. Additionally, clinically meaningful improvements in visual performance were also observed in these short-term supplementation trials between three and six months, respectively [34,116]. Most notably, the randomized, placebo-controlled Diabetes Visual Function Supplement Study (DiVFuSS) demonstrated an average increase of 27% in MPOD levels (measured by HFP) after six months of active supplementation [34]. This study revealed that daily supplementation of 4 mg lutein and 8 mg zeaxanthin plus antioxidants offered significant improvement in contrast sensitivity, color discrimination error score and mean visual field sensitivity in diabetic patients presenting with or without mild-to-moderate NPDR [34]. Thus, these results suggest that carotenoid vitamin therapy formulation may offer protection against diabetes-induced retinal neurodegenerative pathology with concomitant effects on visual performance measures in both type 1 and type 2 diabetes. In fact, the enhanced neuroprotective capacity of a similar carotenoid formula has been shown in experimental model of DR using chemical induction to recapitulate pathology observed in T1DM, discussed previously [34,104,166]. The risk of bias was low for this trial as assessed by the Cochrane Collaboration’s tool.

Various reports seem to suggest these improvements in visual performance following increases in serum carotenoid levels and MPOD concentrations may be attributed, at least in part, to the enhanced functional capacity of the macular pigments to preferentially absorb short-wavelength blue light [27,174,175,176,177,178,179,180,181,182,183]. Greater MPOD levels may provide neuroprotective, pre-receptoral filtration against harmful blue light thereby attenuating the deleterious effects of chromatic aberration [27,178,180,181,182,183]. One school of thought argues that MPOD status may represent a sine qua non for improvements in visual function; namely, that significant benefit in visual performance will occur only after MPOD density has been maintained at greater concentrations for a period of time [62,178,184]. Alternatively, carotenoid vitamin therapy is also believed to augment total antioxidant capacity which may ameliorate intracellular redox homeostasis in the surrounding tissue including the photoreceptor cells of the neurosensory retina [26,127,185,186]. Further implications of greater carotenoid levels in the macula are also thought to improve metabolic efficiency of the visual cycle thereby promoting enhancement of the post-receptoral circuitry [187]. Indeed, the neuroprotective benefits in ganglion cells and photoreceptors observed in experimental models [102,106,107] are also implicated in humans marked by restoring clinical measures of both inner and outer retinal function, respectively [34,116,169,188]. By augmenting their levels in the diet through oral supplementation, the potent antioxidant and anti-inflammatory properties of xanthophyll carotenoids likely counteract the compounding insult from oxidative stress and chronic inflammation in the diabetic retina, as discussed previously [10,55,189,190,191,192,193,194,195,196,197,198,199,200,201,202,203,204]. However, future studies are required in order to elucidate the precise mechanisms responsible for the visual improvements in diabetic retina using carotenoid vitamin therapy.

**Figure 5:**
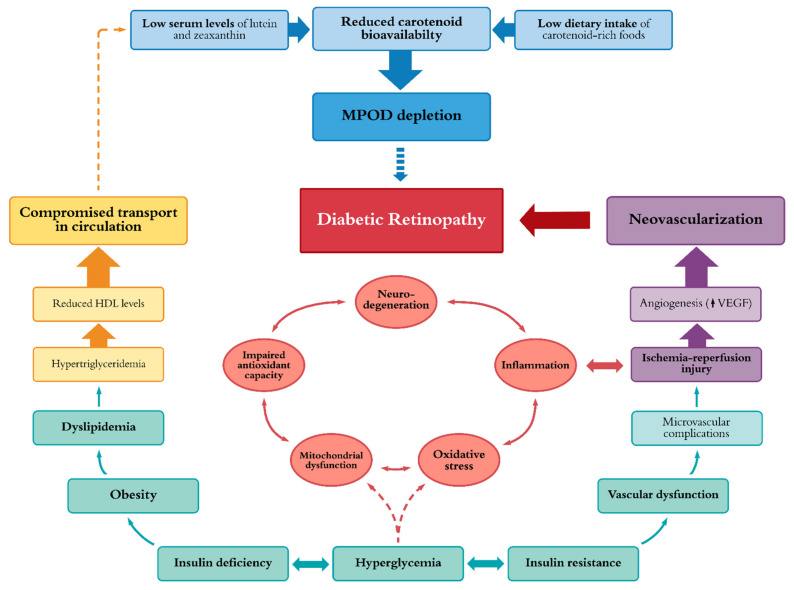
Schematic overview of proposed causative mechanisms and metabolic perturbations implicated in diabetic retinopathy. MPOD, macular pigment optical density; HDL, high-density lipoprotein; VEGF, vascular endothelial growth factor.

In view of these findings, available reports among diabetic patients with and without non-proliferative retinopathy are encouraging in demonstrating the potential for carotenoid supplementation as an adjunct nutraceutical approach to offer enhanced protection against further hyperglycemia-induced injury to the retina. Figure 5 illustrates major causative mechanisms which have been postulated in diabetic retinopathy onset, of which, several interconnected processes are believed to represent key drivers among those with type 2 diabetes or poorly-managed type 1 diabetes [34,205]. One mechanism of action involves systemic, atherogenic metabolic imbalance which is believed to play a significant role in macular pigment depletion [49,189,190,192,195,202,205,206,207,208]. Prior to exerting their nutraceutical effects, lutein and zeaxanthin acquired from the diet must first be released and then absorbed from food matrices before being transported into circulation [56,206,209]. The bioavailability of these dietary xanthophylls in the blood has been shown to fluctuate greatly as a consequence of high-glycemic-index foods [205,206,210,211,212,213]. It is known that dietary behaviors such as those in the Western diet contribute significantly to the onset of metabolic syndrome and may also contribute to MPOD depletion in DR. Thus, metabolic perturbations typically present in patients with T2DM or poorly controlled T1DM, such as obesity, dyslipidemia, insulin deficiency and hyperglycemia are believed to substantially compromise the bioavailability and assimilation of dietary lutein and zeaxanthin to the retina [55,189,190,191,192,193,194,195,206]. The bioavailability of dietary carotenoids is also strongly influenced by age, gender, and racial/ethnic origin, in addition to these anthropometric measures [55,112,175,176,189,190,192,193,194,195,206].

While there are no established recommendations currently regarding daily intake levels of lutein and zeaxanthin consumption, oral supplementation with these carotenoids has a relatively high safety profile, with low risk for adverse effects and are appropriately considered by the US Food and Drug Administration to be Generally Regarded as Safe (GRAS) [214]. Large-scale epidemiological studies are needed to elucidate the putative role of dietary carotenoid intake and risk of DR along with disease progression among cohorts of both type 1 and type 2 diabetic patients. To this point, population data in healthy individuals on dietary intake levels of lutein and zeaxanthin is fairly limited and likely varies significantly among populations based on their dietary behaviors, as mentioned previously [28]. However, one may speculate that individuals whose diet primarily consist of foods rich in refined carbohydrates and artificially sweetened beverages containing high-fructose corn syrup, such as those with T2DM or poorly controlled T1DM for instance, are likely to have significantly lower levels of daily carotenoid intake when compared to those following a Mediterranean-style diet [28,205,215,216,217]. This may be explained, at least in part, by the disparities in regular consumption of various functional food groups (i.e., fresh fruit, nuts, leafy vegetables, and unrefined cereals), of which, several possess relatively high concentrations of lutein and zeaxanthin content per serving (Table 5) [27,28,44,215,218,219]. Based on the available evidence, it remains unclear whether relying solely upon dietary consumption of these carotenoid-rich food is sufficient to achieve the neuroprotective benefits with greater MPOD levels observed in patients with type 1 and type 2 DM following the use of carotenoid vitamin therapy.

It is important to note that these clinically meaningful benefits in diabetic patients with or without DR were independent of any changes in hyperglycemic status or in relation to blood glucose control. Moreover, based on these results, there is a considerable body of preliminary evidence to substantiate the neuroprotective capacity of macular carotenoids to inhibit or reverse disease progression by ameliorating the metabolic correlates and comorbidities often seen in patients with type 2 or poorly controlled type 1 diabetes. Encouraging results from early interventional studies offer scientific justification for renewed clinical trials thereby corroborating the potential use of carotenoid vitamin therapy as an adjunctive therapeutic approach in the management of diabetic retinopathy for patients with either type 1 or type 2 diabetes.

However, there are several limitations currently that must be addressed in future clinical studies should carotenoid supplementation be used for this purpose. First, there is a growing need for further studies to investigate the potential implications associated with long-term use of adjunctive carotenoid vitamin therapy in larger cohorts of individuals with T1DM and T2DM. Second, additional randomized placebo-controlled trials are needed to determine the optimal dosage of lutein and zeaxanthin necessary to achieve clinically meaningful benefits, in addition to whether all three xanthophyll carotenoids found in the retina should be included in formulation. A recent systematic review in healthy adult eyes, found that lutein and zeaxanthin intake of less than 5 mg per day (by oral supplement or food sources) was insufficient dosage to significantly raise MPOD levels during trials up to six months [220]. Additionally, there have been no clinical trials investigating the effects of oral supplementation with *meso*-zeaxanthin in diabetic patients with or without DR to date. Further investigations are required to better understand if the addition of *meso*-zeaxanthin in combination with lutein and zeaxanthin may offer greater benefit or ascertain whether formulations with the two dietary xanthophylls are sufficient to elicit protective effect in diabetic retina. One of the limitations of this systematic review is that the number of databases searched was limited to National Library of Medicine and Web of Science. Additionally, the articles evaluated were limited to those published in the English language.

Lastly, given the systemic etiopathogenesis of diabetes which can manifest in the eye as vascular endotheliopathy, future strategies may focus on ameliorating early microvasculature complications such as retinal vascular occlusion as a consequence of capillary nonperfusion. While experimental models have shown that lutein and zeaxanthin offer protection against retinal capillary degeneration triggered by ischemic-reperfusion injury, it is unclear whether these xanthophylls can prevent microvasculature alterations which ultimately lead to vascular dysregulation. However, oral supplementation with a similar xanthophyll carotenoid known as astaxanthin has been shown in healthy adults has shown to exert benefits on retinal hemodynamic measures including capillary blood flow and velocity of choroidal circulation [221,222,223,224]. Given that astaxanthins retinal uptake has not been clearly demonstrated, its similar neuroprotective properties comparable to those of lutein and zeaxanthin provide scientific rationale for including astaxanthin into carotenoid vitamin therapy formulations in future nutraceutical trials of diabetic retinopathy [221,222,223,224].

## 5. Conclusions

Substantial efforts are necessary in developing early prophylactic measures that offer synergistic protection against several pathogenic mechanisms contributing to retinal neurodegeneration and subsequently preventing irreversible vision loss. To this accord, there is robust preclinical evidence and at least early clinical trials supporting the potential use of carotenoid vitamin supplementation in diabetics with and without retinopathy. Chronic hyperglycemia significantly compromises the endogenous defense systems in a diabetic individual. The metabolic changes due to diabetes possibly lead to depletion of macular carotenoids lutein, zeaxanthin, and *meso*-zeaxanthin, in addition to other potent antioxidants that are pertinent for maintaining retinal health as seen in various observational studies. MPOD measurements may also have a role to play in screening high-risk individuals prior to overt changes in retina due to DR pathology. Further randomized placebo-controlled trials are needed to support and solidify its use more universally as a first line of defense in combination with routine systemic management of diabetes and in susceptible individuals that are at risk of diabetes or pre-diabetics.

## Figures and Tables

**Table 1:** International Clinical Diabetic Retinopathy Disease Severity Scale [37].

Disease Severity Scale	Clinical Features
No apparent retinopathy	No fundus abnormalities present
Mild NPDR	Microaneurysms only
Moderate NPDR	More than just MAs, but less than severe NPDR
Severe NPDR	Any of the following: (with no signs of PDR) extensive DBH in each of 4 quadrants (≥20/quadrants), venous beading in at least 2 quadrants, and/or IRMA in at least 1 quadrant
PDR	One or more of the following: neovascularization, tractional retinal detachment, or vitreous/preretinal hemorrhage

Abbreviations: NPDR, non-proliferative diabetic retinopathy; MA, microaneurysms; PDR, proliferative diabetic retinopathy; DBH, dot blot hemorrhages; IRMA, intraretinal microvascular abnormalities.

**Table 2:** Animal studies of carotenoid treatment in diabetic retinopathy.

Author (Year)	DM Study Design	Duration	Treatment	Results
Arnal (2009) [102]	T1DM, via STZ-injection in Wistar rats	12 wks	L (0.5 mg/kg)	Significantly improved GSH and GPx activity
Kowluru (2008) [103]	T1DM, via STZ-injection in Lewis rats	2 months	Z (8.4 ± 1.6 mg/d); Z (44 ± 8 mg/d)	Enhanced MnSOD and complex III expression
Kowluru (2014) [104]	T1DM, via STZ-injection in Wistar rats	11 months	L (1 mg/d) and Z (2 mg/d) *	Augmented retinal cell viability and survival
Muriach (2006) [105]	T1DM, via A-injection in Albino mice	2 wks	L (0.2 mg/kg)	Re-established levels of MDA, GSH and GPx
Sasaki (2010) [106]	T1DM, via STZ-injection in C57BL/6 mice	4 months	L (0.1% diet)	Protected visual function of inner retina
Tang (2011) [107]	T2DM, via genetic db/db mice (*Lepr*^db^)	8 wks	L (0.05 mg/g fruits) and Z (1.76 mg/g fruits) ^†^	Attenuated ER stress and ganglion cell loss
Yu (2013) [108]	T2DM, via genetic db/db mice (*Lepr*^db^)	8 wks	L and Z (*values not available*) ^†^	Ameliorated hypoxia and mitochondrial stress

Abbreviations: DM, diabetes mellitus; T1DM, type 1 diabetes mellitus; T2DM, type 2 diabetes mellitus; L, lutein; Z, zeaxanthin; STZ, streptozotocin; A, alloxan; db/db, leptin receptor deficient (*Lepr*^db^); GSH, glutathione; GPx, glutathione peroxidase; MnSOD, manganese superoxide dismutase; MDA, malondialdehyde; ER, endoplasmic reticulum * Multivitamin supplement formula; ^†^ Wolfberry nutraceutical.

**Table 3:** A summary of the observational trials.

Author (Year)	Participants	DR Present	Results
Brazionis (2009) [109]	111 patients with T2DM, aged 44–77 years in USA	78 No DR, 33 DR	Lower risk of DR with greater serum levels of non-pro-vitamin A (including L/Z) carotenoids (*p* = 0.039)
Cennamo (2019) [110]	59 patients with T1DM, aged (38.2 ± 13.4) years; 40 healthy controls, aged (31.6 ± 7.4) years in Italy	59 DR	Significantly reduced MPOD (*p* < 0.001) measured by fundus reflectometry
Davies (2002) [111]	34 patients with DM (24 T2DM, 10 T1DM), aged (48.1 ± 11.6) years; 34 healthy controls, aged (36.7 ± 15.1) in United Kingdom	Not specified	Significant lower MPOD among patients with grade 2 maculopathy (*p* = 0.016)
Lima (2010) [112]	29 patients with T2DM, aged (60.7 ± 10.7) years; 14 healthy controls, aged (56.2 ± 11.7) years in USA	17 No DR, 12 NPDR	T2DM patients with or without retinopathy showed reduced MPOD (*p* < 0.001) measured by autofluorescence
Mares (2006) [113]	1698 women from CAREDS, aged 53–86 years (108 patients with diabetes) in USA	Not specified	MPOD measured by HFP (*p* < 0.01) significantly inversely related to diabetes and waist circumference
Scanlon (2015) [19]	102 patients with DM (34 T1DM, 68 T2DM), aged (53.2 ± 12.2) years; 48 healthy controls, aged (52.5 ± 16) years in Ireland	55 No DR, 47 NPDR	MPOD measured by cHFP significantly lower among T2DM (*p* = 0.04) compared to T1DM and controls
Scanlon (2019) [25]	188 patients with T2DM, aged (64.7 ± 8.3) years; 2594 healthy controls, aged (61.4 ± 7.6) years in Ireland	152 No DR, 10 NPDR	T2DM patients saw lower MPOD (*p* = 0.047) measured by cHFP compared to non-diabetic controls
She (2016) [114]	182 patients with DM, aged (62.5 ± 7.2) years; 219 healthy controls, aged (63.6 ± 7.4) years in China	134 No DR, 48 NPDR	MPOD level measured by HFP was significantly associated with central foveal thickness (*p* = 0.001)
Zagers (2005) [115]	14 patients with DM, aged (46 ± 11) years; 14 healthy controls, aged (47 ± 11) years in Netherlands	Not specified	Diabetic eyes showed significant reduction in fundus reflectance MPOD measurement (*p* < 0.001) compared to controls

Abbreviations: NPDR, non-proliferative diabetic retinopathy; DM, diabetes mellitus; T1DM, type 1 diabetes mellitus; T2DM, type 2 diabetes mellitus; L, lutein; Z, zeaxanthin; HFP, heterochromatic flicker photometry; cHFP, customized heterochromatic flicker photometry.

**Table 4:** Characteristics of the eligible randomized clinical trials.

Author (Year)	Participants	DM Subtype	Duration	Interventions	Results
Chous (2016) [34]	67 patients with no retinopathy or mild/moderate NPDR, aged (56.1 ± 13.2) years in USA	27 T1DM, 40 T2DM	6 months	Daily: 4 mg L and 8 mg Z (*n* = 39, multivitamin oral supplementation) ^†^; placebo (*n* = 28)	Significant increase in MPOD (*p* < 0.001), contrast sensitivity (*p* < 0.01, for all) and color error score (*p* < 0.001)
Hu (2011) [116]	60 patients with NPDR, aged (59.5 ± 14.5) years; 30 healthy controls aged (55 ± 9.0) years in China	10 T1DM, 50 T2DM	3 months	Daily: 6 mg L and 0.5 mg Z (*n* = 30 NPDR); placebo oral supplementation (*n* = 30 NPDR, 30 controls)	Significant increase in serum L/Z (*p* < 0.001), visual acuity (*p* < 0.001) and contrast sensitivity (*p* < 0.05, for all)

Abbreviations: NPDR, non-proliferative diabetic retinopathy; T1DM, type 1 diabetes mellitus; T2DM, type 2 diabetes mellitus; L, lutein; Z, zeaxanthin; ^†^ EyePromise DVS multivitamin supplement.

**Table 5:** Common dietary sources of xanthophylls lutein and zeaxanthin [218,219].

Foods	Serving Size	Lutein + Zeaxanthin Content (mg)
Spinach, frozen (cooked)	1 cup	29.8
Kale, frozen (cooked)	1 cup	25.6
Swiss chard (cooked)	1 cup	11.0
Collard greens, frozen (cooked)	1 cup	8.9
Summer squash (cooked)	1 cup	4.0
Peas, frozen (cooked)	1 cup	3.8
Brussel sprouts, frozen (cooked)	1 cup	2.4
Broccoli, frozen (cooked)	1 cup	2.0
Edamame, frozen	1 cup	1.6
Sweet yellow corn (boiled)	1 cup	1.5
Asparagus (boiled)	0.5 cup	0.7
Avocado, raw	1 medium-size	0.4
Egg yolk, raw	1 large	0.2
